# Structural Analysis of a Genetically Encoded FRET Biosensor by SAXS and MD Simulations

**DOI:** 10.3390/s21124144

**Published:** 2021-06-16

**Authors:** Ines Reinartz, Mona Sarter, Julia Otten, Henning Höfig, Martina Pohl, Alexander Schug, Andreas M. Stadler, Jörg Fitter

**Affiliations:** 1 Institute for Automation and Applied Informatics, Karlsruhe Institute of Technology, Hermann-von-Helmholtz-Platz 1, 76344 Eggenstein-Leopoldshafen, Germany; ines.reinartz@kit.edu; 2 HIDSS4Health-Helmholtz Information and Data Science School for Health, 76344 Eggenstein-Leopoldshafen, Germany; 3 I Physikalisches Institut (IA), AG Biophysik, RWTH Aachen University, 52074 Aachen, Germany; mona.sarter@stfc.ac.uk (M.S.); h.hoefig@fz-juelich.de (H.H.); 4 Forschungszentrum Jülich, IBI-8/JCNS-1, 52428 Jülich, Germany; a.stadler@fz-juelich.de; 5 Forschungszentrum Jülich, IBG-1, 52426 Jülich, Germany; j.otten@fz-juelich.de (J.O.); ma.pohl@fz-juelich.de (M.P.); 6 Forschungszentrum Jülich, IBI-6, 52428 Jülich, Germany; 7 John von Neumann Institute for Computing, Jülich Supercomputing Centre, Forschungszentrum Jülich, 52428 Jülich, Germany; al.schug@fz-juelich.de; 8 Faculty of Biology, University of Duisburg-Essen, 45141 Essen, Germany; 9 Institut für Physikalische Chemie, RWTH Aachen University, 52074 Aachen, Germany

**Keywords:** glucose sensor, green fluorescence protein (GFP), single-molecule FRET, small angle X-ray scattering (SAXS), coarse-grained molecular dynamics (MD)

## Abstract

Inspired by the modular architecture of natural signaling proteins, ligand binding proteins are equipped with two fluorescent proteins (FPs) in order to obtain Förster resonance energy transfer (FRET)-based biosensors. Here, we investigated a glucose sensor where the donor and acceptor FPs were attached to a glucose binding protein using a variety of different linker sequences. For three resulting sensor constructs the corresponding glucose induced conformational changes were measured by small angle X-ray scattering (SAXS) and compared to recently published single molecule FRET results (Höfig et al., *ACS Sensors*, 2018). For one construct which exhibits a high change in energy transfer and a large change of the radius of gyration upon ligand binding, we performed coarse-grained molecular dynamics simulations for the ligand-free and the ligand-bound state. Our analysis indicates that a carefully designed attachment of the donor FP is crucial for the proper transfer of the glucose induced conformational change of the glucose binding protein into a well pronounced FRET signal change as measured in this sensor construct. Since the other FP (acceptor) does not experience such a glucose induced alteration, it becomes apparent that only one of the FPs needs to have a well-adjusted attachment to the glucose binding protein.

## 1. Introduction

Important representatives of biosensors are molecular structures that link highly specific ligand-binding properties of biological macromolecules, mainly proteins, to a physical observable, which can be quantified by a read-out measurement [1,2,3]. In particular, periplasmic binding proteins (PBPs) offer a large potential, since they provide an allosteric linkage between binding of an effector ligand and a pronounced large-scale hinge-bending movement of two globular domains [4,5]. In the case of genetically encoded Förster resonance energy transfer (FRET)-based sensors, the sensors are often constructed as fusions of full-length protein chromophores, typically green fluorescent protein variants of different colors, attached via short linkers to the ligand binding protein termini. Based on known crystal structures of several PBPs, the distance in the range of 4–5 nm between N and C termini is well suited to enable Förster resonance energy transfer (FRET) between the two attached fluorescent proteins (FPs) acting as a donor/acceptor pair [5,6]. Importantly, the FRET transfer efficiency can vary significantly upon small changes in distance or orientation between the fluorescent proteins, caused by the ligand-induced hinge-bending movement of the ligand binding protein. Resulting changes in the fluorescence intensity of the donor and acceptor FP can be utilized as an optical read out to determine ligand concentrations in the sensor environment [5,6,7,8]. The question, to which extent good sensors make use of changes, either in distance or in relative orientation of the involved FPs (or a combination of both), is still under debate [7,9,10]. In this respect, further methodical approaches to gain relevant structural details of sensor constructs are provided directly by small-angle X-ray scattering (SAXS) studies [11,12,13] complemented by rigid-body modelling and molecular dynamics simulations [14,15]. In addition to sensor platforms, which employ unimolecular conformational FRET sensors (i.e., a single sensing protein flanked by two FPs), further types of fluorescence-based sensors have recently been developed. These sensor types include fluorescence modulated single FP-based sensors, complementation sensors, cleavage FRET sensors, or dimerization-based sensors [3,16,17].

Furthermore, we performed a combined analysis to characterize ligand induced conformational changes of three different constructs of a recently developed FRET-based glucose biosensor [18]. These constructs are based at the glucose-galactose binding protein from *Escherichia coli* (MglB) flanked by two FPs and make use of the Venus flytrap principle [7]. Since the three constructs differ significantly in their FRET intensity signal change upon glucose binding (sensor sensitivity), we investigated the corresponding conformational changes with different complementary approaches. First, SAXS measurements with the sensor constructs under ligand-free and ligand-saturated conditions were performed. Furthermore, we made use of recently acquired single molecule FRET data [18] and additionally conducted coarse-grained molecular dynamics simulations on the sensor construct with the best sensing performance. The aim of this combined analysis was to investigate how structural arrangements of the FPs change upon ligand binding and how this change is correlated with the change of the FRET transfer efficiency.

## 2. Materials and Methods

### 2.1. Sensor Constructs

Based on the FLII^12^P-glu 600 µ construct by Frommer and co-workers [7], we recently generated a sensor toolbox with different linker sequences flanking the central glucose-galactose binding protein from *E. coli* (MglB) and mTurquoise2 and Venus as the FRET pair [19]; see also Supplementary Materials Section S1. In this study, sensor constructs no. 1 and 4 were used as representatives for weak or only moderate sensor performance, while construct no. 2 represents a very efficient sensor [18]. For details about the chosen constructs, see Supplementary Materials, Figure S1.

### 2.2. SAXS Measurements and Data Analysis

Small-angle X-ray scattering (SAXS) experiments were performed on glucose sensor constructs, which were synthesized and purified as described earlier [18,19]. Since the glucose sensor constructs tend to form aggregates when concentrated to a mg/mL range, a size exclusion chromatography (SEC)-SAXS approach was employed in the present study [20]. After the SEC column (Superdex 200 Increase 10/300 GL) was equilibrated with buffer (20 mM MOPS, 150 mM NaCl in H_2_O, pH 7.3), 100 μL of the respective glucose sensor construct was added to the column at a concentration of 5 mg/mL and 10 mg/mL via a 100 µL loop. The three sensor constructs (nos. 1, 2, 4) were measured in the absence of glucose and under glucose saturated conditions (100 mM glucose in the buffer). Samples were diluted by a factor of around 1.4 during the SEC run. SAXS measurements were performed at the BM29 BIOSAXS beamline (ESRF, Grenoble, France) using a wavelength of 0.992 Å [21]. SAXS data were recorded continuously during the SEC run and binned into data frames with a 1 s exposure time per frame. We used an automated data analysis pipeline protocol to select the corresponding X-ray frames of the monomeric SEC elution peak and of the pure buffer scattering. Data frames were automatically checked for the absence of protein aggregation, for radiation damage and merged. Buffer scattering was subtracted from the merged data frames and buffer-subtracted data was normalized by the measured protein concentration. Finally, the normalized SAXS data of the 5 mg/mL and 10 mg/mL protein solutions were compared and no effects due to protein-protein interactions were found. Therefore, normalized SAXS data of both concentrations were merged and used for further data analysis. Temperature at the sample position was 293 K during the experiment, the used X-ray detector was a Pilatus 1M and sample to detector distance was 2.869 m. Further data analysis was performed using the algorithms available within the ATSAS software package [22]. The distance distribution function *P*(*r*) and the Porod volume of the protein were determined with the programs, GNOM and DATPOROD, respectively. Guinier analysis was performed in a *q*-range with *qR*_G_ < 1.3 yielding the Guinier radius *R*_G_ and the forward scattering *I*(0). Maximal dimension of the particle *D*_max_ and Guinier radii in real *R*_G,real_ and reciprocal space *R*_G,rec_ were determined from the *P*(*r*) function. The molecular weight was calculated from the Porod volume as described by Petoukhov et al. [23] using a proportionality factor of 0.625. In addition, the molecular mass was also determined using Bayesian inference based on SAXS different invariants as described by Hajizadeh et al. [24].

### 2.3. Modelling and Simulations

We simulated sensor construct no. 2 with the simulation framework of structure-based models (SBMs) [25], implemented in eSBMTools [26] to accommodate for large-scale dynamics and significant time scales. Based on energy landscape theory and the principle of minimal frustration, SMBs are a coarse-grained MD variant widely applied for either large, long, or both, timescale simulations [27]. For the simulations, a starting structure of atomic detail as well as parameters for the system were required. Based on the sensor sequence (see Supplementary Materials, Section S1) we first modeled a structure of the sensor construct by attaching the different structural parts successively. For the glucose/galactose binding protein (MglB) and the FPs we used the respective PDB structures (MglB: 2FVY and 2FW0 [4], mTurquoise2: 3ZTF [28], Venus: 1MYW [29]). Since there is no determined three-dimensional structure for the linkers, only limited information about their structural and dynamic properties was available. To incorporate the linkers into the simulations, we generated structures based on their sequence. This generation of structures resulted in a wide ensemble of possible structures for the sensor construct. To narrow the conformational space for the simulations, we fitted the resulting structures to the respective experimental SAXS data of the sensor in the glucose bound state using CRYSOL [30]. As a starting point for the simulations, we chose the four best fitting structures with the lowest χ^2^ values (see Section 3.2). We parametrized proteins and linkers similar to dye-labeled proteins [31]. All simulations of the sensor construct were performed with GROMACS v4.5.4 [32] with the extension for Gaussian contact potentials [31], Langevin dynamics and an SBM potential; see [31,33]. In the simulations, the linker was left rather flexible in comparison to the rest of the structure, to account for its flexibility and the uncertainty regarding its structure. As SBMs do not have an inherent time scale, we determined the time scale by comparison of rotational correlation times of free FPs between simulations and experimental values. For the evaluation, we generated SAXS intensity profiles of structures distributed evenly over the entire simulation using CRYSOL [30,34]. To compare the rotational flexibility of both FPs in the sensor, we calculated the fluorescence anisotropy and the rotational correlation times of the FPs bound to MglB. By fitting the simulated trajectory of MglB, we could observe the FP motions independently of the overall rotation of the system, which is experimentally hard to achieve. Due to the restriction of the rotational motions, we used the wobbling-in-a-cone model, where the anisotropy decay is described by the rotational correlation time, the fundamental anisotropy and the angle of the cone [35]. More details about the structures, parameters, simulation procedure and analysis are given in the Supplementary Materials, Section S2.

## 3. Results and Discussion

### 3.1. Comparison of Single Molecule FRET and SAXS Results

The sensor constructs used here were identified from a previously developed sensor toolbox [18,19]. As already stated by Deuschle and co-workers, a good sensor design is closely related to establishing a strong allosteric linkage between the ligand-induced conformational change in the glucose/galactose binding protein (MglB) and the chromophore rearrangement [7]. In that respect, short linkers connecting the MglB with each of the FPs must be chosen in a way that (i) the distance between both FPs remains close enough to observe FRET changes and (ii) the rotational freedom of one or both FPs should be reduced in order to obtain a better coupling of conformational change to FRET change. Therefore, the sensors were constructed with the donor FP inserted between positions 12 and 13 at the N-terminal end of the MglB sequence, thereby establishing a tighter link of the donor FP to the binding region [7].

**Figure 1:**
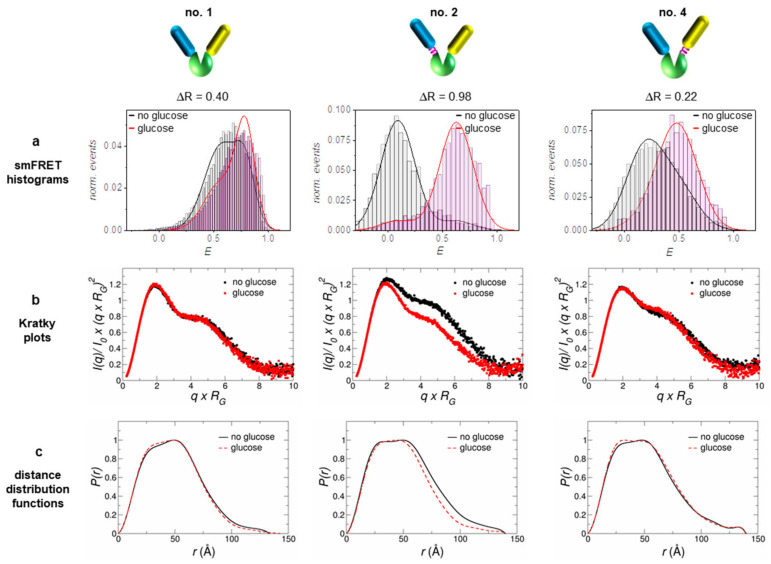
The three investigated sensor constructs exhibit structural differences with respect to how the FPs are attached to the glucose binding protein MglB: no linker between MglB and both FPs (no. 1, left), a flexible linker between MglB and the donor FP (no. 2, middle) and a flexible linker between MglB and the acceptor FP (no. 4, right). The corresponding ΔR-values were determined from ensemble Förster resonance energy transfer (FRET) measurements (FRET binding isotherms, see [18]). (**a**) Data from single molecule measurements (smFRET histograms) were taken from an earlier publication, where these constructs were analyzed in detail [18]. Based on the analysis of the measured small angle X-ray scattering (SAXS) data, the respective Kratky plots (**b**) and distance distribution functions (**c**) are presented in the two lower lines. For further details, refer to the Supplementary Materials, Figures S3.2 and S3.3, and Table S2.

In addition to the sensor prototype without linkers (no. 1), one construct with a short flexible linker at the N-terminal FP (no. 2) and another one with a flexible linker at the C-terminal FP (no. 4) were characterized by SAXS measurements (for corresponding sequences see Supplementary Materials, Section S1). The respective individual sensor sensitivities, measured by the FRET signal changes for these constructs, were characterized in detail earlier [18] and the corresponding results are summarized in Figure 1 and Table 1. The sensitivity of a sensor construct is determined by measuring the ratio of acceptor and donor fluorescence emission (R = I_A_/I_D_) as a function of ligand (glucose) concentration. Effectively, the obtained R-value is a measure of the Förster resonance energy transfer which changes upon ligand binding. The resulting sigmoidal shaped binding isotherms yield the corresponding apparent dissociation constant and the effective sensitivity of the sensor construct, given by ΔR (see [18]). The latter displays the difference between R-values in absence of the ligand (R_min_) and R-values saturated with ligand (R_max_). The K_d_-values for D-glucose of the investigated constructs range between a few and a few tens of mM, while above 100 mM all constructs are fully saturated with glucose.

In order to obtain a more detailed picture about how a reasonable sensor sensitivity is achieved, we used single molecule FRET data from earlier measurements; for details see [18]. In accordance with the obtained ΔR-values, the population shift ΔE is most pronounced in the case of sensor no. 2 (see Figure 1a and Table 1). Only for construct no. 2 we observed two well-separated populations for each state, with an almost complete (~90%) transfer from one state to the other upon ligand binding/release. Compared to this, the other two constructs show a significantly smaller shift between the populations, where that of construct no. 1 is still smaller when compared to that of construct no. 4 (see Table 1).

To obtain direct structural information on the sensor constructs, we performed SAXS measurements with the constructs in the glucose-bound state (displayed in red) and the glucose-free state (displayed in black); see Figure 1b,c. The Kratky plots as well as the distance distribution functions clearly discriminate construct no. 2 from the other two. Only sensor no. 2 displays a pronounced structural compaction upon glucose binding in the respective plots. This fact is also supported by the obtained R_G_-values from a Guinier analysis given in Table 1.

**Figure 2:**
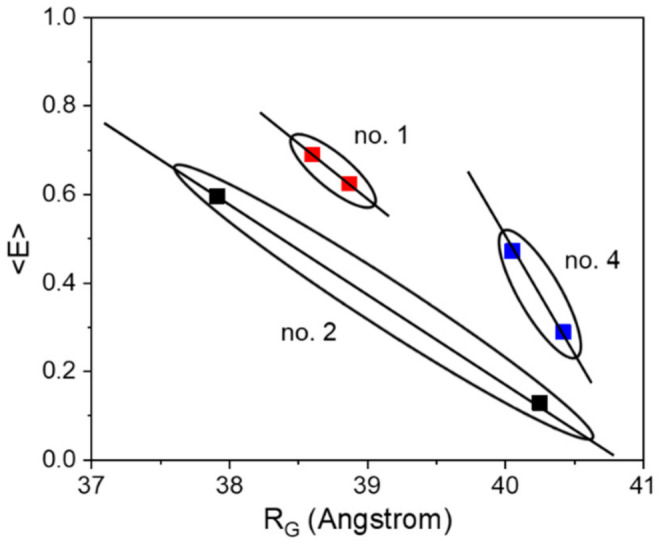
The correlation between energy transfer change and change in R_G_ is shown for the three investigated sensor constructs. Although we do not expect a strict linear relationship between <E> and R_G_, the solid lines do not represent fits, but should visualize a general trend.

Nevertheless, the other sensor constructs also exhibit a small compaction upon ligand binding, although smaller by a factor of 6–9 (in terms of ΔR_G_) as compared to construct no. 2. Qualitatively, all sensor constructs follow the same structural behavior of the pure glucose binding protein MglB, which also shows a distinct compaction upon glucose binding (see Supplementary Materials, Figure S3.1). Although the radius of gyration R_G_ is by definition not equal to the distance R_DA_ between both FPs (relevant FRET parameter), the change in R_G_ (ΔR_G_) and the change in energy transfer ΔE appear to be highly correlated (see Table 1 and Figure 2). Both corresponding difference values are the largest for sensor construct no. 2, followed by construct no. 4, while construct no. 1 displays the smallest difference. Without knowing the relative orientation of the involved FPs (i.e., the respective transition dipole moments; see for example [36]), a direct measure of the inter-FP-distance change ΔR_DA_ upon substrate binding is not achievable. However, by looking at the ΔE/ΔR_G_ ratio (slope of solid lines in Figure 2) we can estimate to which extent the individual sensor construct makes use of a sensor compaction in order to achieve a certain change in transfer efficiency (i.e., sensor read out signal).

### 3.2. Modelling and Coarse-Grained Molecular Dynamics Simulations of Sensor Construct no. 2

**Figure 3:**
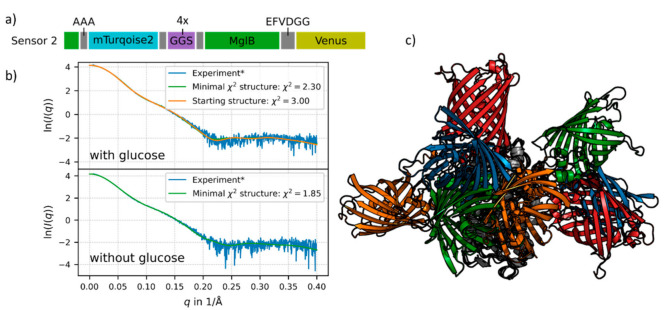
(**a**) Schematic presentation of the sensor no. 2 sequence. The flexible linker (purple) and restriction sites (grey) are shown with their respective sequences. The donor FP (mTurquoise2, cyan) is inserted into MglB (green), so the first 11 residues of MglB are in the beginning of the sequence and the main part in the middle. The acceptor FP (Venus) is shown in yellow (for details see Supplementary Materials, Section S1). (**b**) The experimental SAXS data is shown in blue, the fit of the chosen starting structure (blue FPs in Figure 3c) in orange, the latter is only shown for the glucose-saturated structure. Structures from the simulations with respective minimal χ^2^ values are shown in green. (**c**) Starting structures for the simulation of sensor no. 2 with MglB in the glucose-saturated state. All structures are aligned to MglB (grey). The different pairs of fluorescent proteins are depicted in orange (sensor 2-1, χ^2^ = 3.0), blue (sensor 2-2, χ^2^ = 3.11), green (sensor 2-3, χ^2^ = 3.75) and red (sensor 2-4, χ^2^ = 4.1). For details see Supplementary Materials, Section S2.6.

To obtain a more detailed understanding about the working principles of the FRET sensor constructs on a molecular level, rigid-body modelling and coarse-grained molecular dynamics simulations were performed for construct no. 2. Since the latter exhibits the highest sensor sensitivity in terms of FRET signal change, we chose this construct for simulation studies. For this purpose, we first prepared rigid body models with two distinct MglB crystal structures, one with ligand bound and one without. The comparison of both structures’ stresses exhibits a significant conformational change upon glucose binding which is characterized by a pronounced hinge-twist motion of the two domains against each other [4]. Due to the ability to transfer the MglB conformational change into a large FRET-ratio change, the flexible linker between MglB and the donor FP of the sensor construct no. 2 appears to be crucial for the superior performance of this sensor (see sequence of this construct in Figure 3a).

The merging of the sensor construct no. 2 resulted in a wide ensemble of possible structures. We fitted the resulting structures to experimental SAXS data and chose four different starting structures for the glucose-bound state (see Figure 3c) which were selected based on the lowest χ^2^-values (see Figure 3b and legend of Figure 3). Further simulations with these different starting structures were performed for the glucose-saturated and in the glucose-free state. Each simulation represents the dynamics of the system during approximately 100 μs on the physical time scale (for methodical details of the simulation see Supplementary Materials, Section S2).

For the glucose-bound state of one sensor no. 2 starting structure (orange FPs in Figure 3c, orange in Figure 3b) we exemplarily show a comparison of the simulated data with experimental SAXS data. The approach of using different starting structures in our simulations helped to cover the large conformational space of the flexible sensor constructs. Structures from the simulations showing the best concordance with the experimental SAXS data are shown in green in Figure 3b. In the case of the ligand-saturated state, this is very similar to the starting structure (Figure 3b, orange fit). The obtained χ^2^ values describing the deviation of the mean intensity curve of the simulation with respect to the experimental SAXS data are quite small, for the glucose-free state (lower panel in Figure 3b) as well as for the glucose-saturated state (upper panel in Figure 3b).

**Figure 4:**
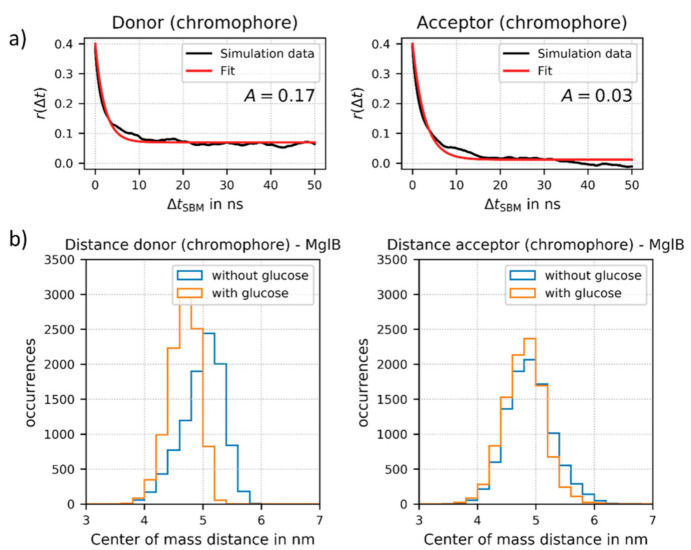
(**a**) Fits of the fluorescence anisotropy r(Δt) as a function of time Δt_SBM_ for the donor chromophore (as part of mTurquoise2) and for the acceptor chromophore (as part of Venus) attached to MglB (here in the glucose-bound state). The time Δt_SBM_ is given in ns in the SBM simulation, which does not directly correspond to a physical time scale. The model function for the “wobbling-in-a cone” model (solid red) was fitted to r(Δt) values obtained from the simulation of sensor no. 2. The respective rotational correlation times on a physical time scale are in a time regime of 190–290 ns (further details see Supplementary Materials, Section S2). (**b**) Relative distance change upon glucose binding between the donor FP and the center of the MglB (left) and between the acceptor FP and the MglB (right), respectively. For the distance calculation we used the center of mass of the respective chromophore of the FPs.

The simulation trajectories were then utilized to analyze the rotational mobility of each of the attached FPs (for details see Supplementary Materials, Section S2.9). For this purpose, we calculated the time-dependent fluorescence anisotropy and the rotational correlation times of the chromophores (as part of the FPs) bound to MglB. This approach allowed obtaining the motion of the fluorophore independently of the overall sensor construct rotation, in contrast to the experimental time resolved anisotropy measurements. Two exemplary fits are shown in Figure 4a. Due to possible restrictions of the FP motion, the “wobbling-in-a-cone” model [35] is more appropriate here than a simple exponential fit. This type of analysis is well suited to determine approximations of rotational correlation times of the fluorescent proteins in the sensor, as they are not accessible directly by the conducted experiments. The derived values for rotational correlation times for the donor FP and the acceptor FP in the sensor construct are on a physical time scale of 190–290 ns.

This indicates that the FPs are still mobile, but on a much longer time scale as compared to, for example, free fluorescent protein (correlation time of about 20 ns). The rotational correlation times of the attached fluorophores are also much longer than the fluorescence lifetimes of both fluorophores (a few ns). Therefore, the normally applied approach of isotropic dynamic averaging (resulting in a κ^2^-value of 2/3) cannot be applied. Importantly, these results support the assumption that for improved sensor design, the rotational freedom of at least one FP should be reduced. Based on our results, the rotational mobility of the inserted donor FP at the N-terminal end of construct no. 2 is geometrically hindered. This conclusion is supported by a relatively large amplitude factor A (or large r(t) at long Δt_SBM_) as shown for the ligand bound state in Figure 4a. Due to this non-isotropic donor FP rotation in the ligand-bound state, we could not calculate reliable κ^2^ and R_DA_ values from the experimental FRET data, which excludes a direct comparison between experimentally determined and simulated inter FP distances in the present. In contrast, the acceptor FP is fused directly to the C-terminal end of MglB and does not show such restrictions (see small A-value in Figure 4a). Additionally, the donor FP regains flexibility, i.e., a decrease in A-values, in structures without glucose, whereas this change is negligible for the acceptor FP (see Supplementary Materials, Section S2.9).

Finally, we also analyzed the changes in distance between the donor FP and the center of the MglB structure and between the acceptor FP and the MglB, respectively. We could demonstrate that during the simulations the distance between the donor FP and MglB displays a considerable change upon glucose binding (see Figure 4b). Therefore, our simulations further support an important prerequisite of the ideal sensor design, a strong allosteric coupling achieved by inserting the donor FP after position 12 at the N-terminal end of the MglB sequence (see Supplementary Materials, Section S1). In contrast, the distance between the acceptor FP and MglB is not altered significantly upon glucose binding.

## 4. Conclusions

One key in FRET-based biosensor development is to understand and to make use of the structure-function relationship of the sensor constructs which are typically artificial man-made multi-modular fusion proteins. Therefore, complementary to the required FRET measurements, additional methodical approaches like SAXS, NMR, X-ray crystallography and rigid body modelling were already employed in the past to unravel the structure-function relationship [9,11,12,37]. In our approach, we additionally propose the use of single molecule FRET measurements, SAXS experiments and coarse-grained molecular dynamics simulations [31,38].

As demonstrated by the presented SAXS measurements, only a sensor with pronounced conformational change upon glucose binding (sensor no. 2) shows a large change in FRET efficiency which is necessary for a high sensitivity. Although the other two analyzed constructs (no. 1 and no. 4) experienced a similar ligand-induced conformational change of the central binding protein, the resulting conformational changes of the corresponding whole constructs were much less pronounced. As a consequence, the constructs no. 1 and no. 4 show a much smaller, but still measurable, FRET signal change. In summary, we can conclude that only a pronounced, ligand-induced conformational change leads to a considerable sensor sensitivity.

These findings were supported by coarse-grained molecular dynamics simulations. Even though simulations of sensor no. 2 showed relatively large conformational flexibility for the ligand-bound and ligand-free state, sampled within a 100 μs time window, we obtained evidence for a pronounced difference in the mean inter FP distance between the ligand-free and the ligand bound-state (see Supplementary Materials, Figure S2.6). Furthermore, we obtained donor FP-MglB distances values for sensor no. 2 that also show a considerable difference between the ligand-free and ligand-bound state.

Despite this flexibility, the simulation also revealed a reduced rotational mobility of the donor FP due to spatial constrains such as its restricted distance to MglB. In contrast, the acceptor mobility is not hindered. All observed changes between both states are clearly supported by the SAXS data. Our example indicates that, for a good FRET-based sensor, a tight allosteric coupling to at least one of the two attached FPs is sufficient.

## Figures and Tables

**Table 1:** Comparison of structural parameters for the different sensor constructs.

	**No. 1**	**No. 1** **+ Glucose**	**No. 2**	**No. 2** **+ Glucose**	**No. 4**	**No. 4** **+ Glucose**
R_G_ (Å) Guinier	38.87 ± 0.06	38.60 ± 0.08	40.25 ± 0.11	37.91 ± 0.07	40.42 ± 0.10	40.05 ± 0.08
<E> ^1^	0.624 ± 0.023	0.690 ± 0.020	0.129 ± 0.040	0.596 ± 0.040	0.289 ± 0.040	0.472 ± 0.040
ΔR_G_ (Å) ^2^	0.27 ± 0.14		2.34 ± 0.18		0.37 ± 0.18	
ΔE ^3^	0.066 ± 0.043		0.467 ± 0.08		0.183 ± 0.08	
ΔE/ΔR_G_ (Å^−1^)	0.244		0.199		0.459	
ΔR	0.40		0.98		0.22	

^1^ Obtained mean position of the individual population in the smFRET histogram (see Figure 1a). ^2^ Difference between glucose-saturated and glucose-free states calculated based on the R_G_ values. ^3^ Shift of the weighted mean position of the glucose-bound and glucose-free populations as calculated in a previous publication; see [18].

## Data Availability

Not applicable.

## Supplementary Materials

The following are available online at https://www.mdpi.com/article/10.3390/s21124144/s1, Documents S1. Sensor constructs and experimental sequences; S2. Simulations of biosensors with fluorescent proteins; S3. SAXS results; Figures S1.1, S2.1–S2.8, S3.1–S3.3; Tables S1 and S2 (PDF).
