# Supplementary material for: Structural Analysis of a Genetically Encoded FRET Biosensor by SAXS and MD Simulations
Source: Sensors (Basel). 2021 Jun 16;21(12):4144. doi: 10.3390/s21124144 (PMC8234384; doi:10.3390/s21124144)
Supplement: Supplementary file 1 [file sensors-21-04144-s001.zip › sensors-1241277-supplementary.pdf]

# Supporting Information to: Structural Analysis of a Genetically Encoded FRET Biosensor by SAXS and MD simulations

I. Reinartz[1,2], M. Sarter[3,4], J. Otten[5], H. Höfig[3,6], M. Pohl[5], A. Schug[7,8], A. M. Stadler[4,9], and J. Fitter[3,6]

[1]Institute for Automation and Applied Informatics, Karlsruhe Institute of Technology, Hermann-von-Helmholtz-Platz 1, 76344 Eggenstein-Leopoldshafen, Germany
[2]HIDSS4Health - Helmholtz Information and Data Science School for Health, Karlsruhe/Heidelberg, Germany
[3]RWTH Aachen University, I Physikalisches Institut (IA), AG Biophysik, 52074 Aachen, Germany
[4]Research Centre Jülich, IBI-8/JCNS-1, 52428 Jülich, Germany
[5]Research Centre Jülich, IBG-1, 52428 Jülich, Germany
[6]Research Centre Jülich, IBI-6, 52428 Jülich, Germany
[7]Institute for Advanced Simulation, Jülich Supercomputing Center, 52428 Jülich, Germany
[8]Faculty of Biology, University of Duisburg-Essen, Germany
[9]RWTH Aachen University, Institut für Physikalische Chemie, 52074

## Contents

**S1 Sensor Constructs and Experimental Sequences** **2**
S1.1 Sensor Constructs . . . 2
S1.2 Experimental Sequences . . . 3

**S2 Simulation of Biosensors with Fluorescent Proteins** **4**
S2.1 Sensor Sequence for Simulation . . . 4
S2.2 Protein Structures . . . 4
S2.3 Protein Parameters . . . 5
S2.4 Linker Structures . . . 5
S2.5 Linker Parameters . . . 6
S2.6 Merging of Sensing Protein and Fluorescent Proteins . . . 6
S2.7 Simulation Protocol for Fluorescent Proteins . . . 7
S2.8 Analysis of Simulations . . . 10
S2.9 Flexibility of Fluorescent Proteins . . . 12

**S3 SAXS results** **14**

# S1 Sensor Constructs and Experimental Sequences

## S1.1 Sensor Constructs

Based on results of a recent study [1], we selected three out of nine possible constructs for our present study (see Fig. S1.1).

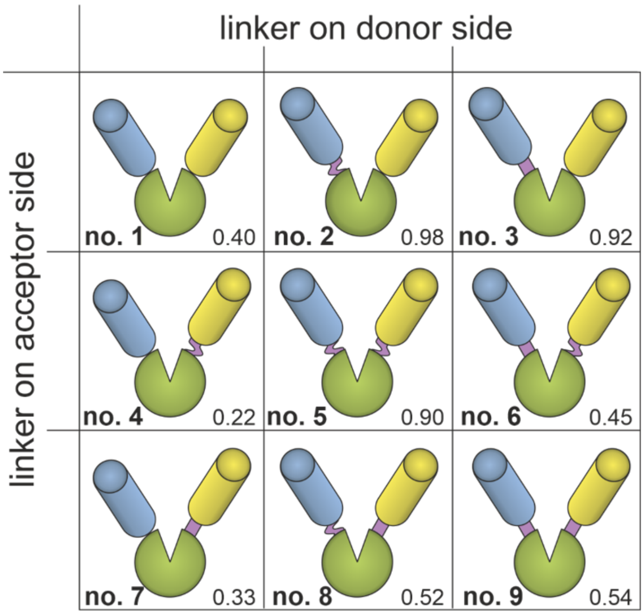


*Figure S1.1: Sensor constructs designed by using the toolbox approach as described in detail in [1]. The additional numbers given in each panel represent the obtained $\Delta R$-values from ensemble FRET measurements. Obviously construct no. 1 and no. 2 exhibit most pronounced sensor sensitivity. In contrast construct no. 4 shows a very low sensitivity, while no. 1 (as well as many other constructs) exhibit an intermediate sensitivity. Therefore, construct no. 1, no. 2 and no. 4 were chosen for the comparative analysis performed in this study.*

## S1.2 Experimental Sequences

The coding sequence for mTurquoise2, the glucose-galactose binding protein (MglB) and Venus are colored in cyan, green, and yellow, respectively. Sequences for the flexible $(GGS)_4$ linkers are colored in purple. Restriction (including linkers) sites are shown in red, and the N- or C-terminal $His_6$-tags are depicted in grey, while linker between Terminal His-tag und construct is depicted in dark grey.

(A): Amino acid sequence of sensor no. 1 with N-terminal His-Tag, no linker.

```
MRGSHHHHHHGMASMTGGQQMGRDLYDDDDKEPGRADTRIGVTIYKAAAMVSKGEELFTGVVPILVELDGDVNGHKFSV
SGEGEGDATYGKLTLKFICTTGKLPVPWPTLVTTLSWGVQCFARYPDHMKQHDFFKSAMPEGYVQERTIFFKDDGNYKT
RAEVKFEGDTLVNRIELKGIDFKEDGNILGHKLEYNYFSDNVYITADKQKNGIKANFKIRHNIEDGGVQLADHYQQNTP
IGDGPVLLPDNHYLSTQSKLSKDPNEKRDHMVLLEFVTAAGITLGMDELYGSDLVDNFMSVVRKAIEQDAKAAPDVQLL
MNDSQNDQSKQNDQIDVLLAKGVKALAINLVDPAAAGTVIEKARGQNVPVVFFNKEPSRKALDSYDKAYYVGTDSKESG
IIQGDLIAKHWAANQGWDLNKDGQIQFVLLKGEPGHPDAEARTTYVIKELNDKGIKTEQLQLDTAMWDTAQAKDKMDAW
LSGPNANKIEVVIANNDAMAMGAVEALKAHNKSSIPVFGVDALPEALALVKSGALAGTVLNDANNQAKATFDLAKNLAD
GKGAADGTNWKIDNKVVRVPYVGVDKDNLAEFSKKEFVDGGMVSKGEELFTGVVPILVELDGDVNGHKFSVSGEGEGDA
TYGKLTLKLICTTGKLPVPWPTLVTTLGYGLQCFARYPDHMKQHDFFKSAMPEGYVQERTIFFKDDGNYKTRAEVKFEG
DTLVNRIELKGIDFKEDGNILGHKLEYNYNSHNVYITADKQKNGIKANFKIRHNIEDGGVQLADHYQQNTPIGDGPVLL
PDNHYLSYQSALSKDPNEKRDHMVLLEFVTAAGITLGMDELYK
```

(B): Amino acid sequence of the sensor no. 2 with N-terminal (of MglB) flexible linker ($(GGS)_4$) and C-terminal His-tag.

```
MADTRIGVTIYKAAAMVSKGEELFTGVVPILVELDGDVNGHKFSVSGEGEGDATYGKLTLKFICTTGKLPVPWPTLVTT
LSWGVQCFARYPDHMKQHDFFKSAMPEGYVQERTIFFKDDGNYKTRAEVKFEGDTLVNRIELKGIDFKEDGNILGHKLE
YNYFSDNVYITADKQKNGIKANFKIRHNIEDGGVQLADHYQQNTPIGDGPVLLPDNHYLSTQSKLSKDPNEKRDHMVLL
EFVTAAGITLGMDELYGSDLVDNFMSVVRKAIEQDAKAAPDVQLLMNDSQNDQSKQNDQIDVLLAKGVKALAINLVDPA
AAGTVIEKARGQNVPVVFFNKEPSRKALDSYDKAYYVGTDSKESGIIQGDLIAKHWAANQGWDLNKDGQIQFVLLKGEP
GHPDAEARTTYVIKELNDKGIKTEQLQLDTAMWDTAQAKDKMDAWLSGPNANKIEVVIANNDAMAMGAVEALKAHNKSS
IPVFGVDALPEALALVKSGALAGTVLNDANNQAKATFDLAKNLADGKGAADGTNWKIDNKVVRVPYVGVDKDNLAEFSK
KEFGGSGGSGGSGGSVDGGMVSKGEELFTGVVPILVELDGDVNGHKFSVSGEGEGDATYGKLTLKLICTTGKLPVPWPT
LVTTLGYGLQCFARYPDHMKQHDFFKSAMPEGYVQERTIFFKDDGNYKTRAEVKFEGDTLVNRIELKGIDFKEDGNILG
HKLEYNYNSHNVYITADKQKNGIKANFKIRHNIEDGGVQLADHYQQNTPIGDGPVLLPDNHYLSYQSALSKDPNEKRDH
MVLLEFVTAAGITLGMDELYKHHHHHH
```

(C): Amino acid sequence of the sensor no. 4 with C-terminal (of MglB) flexible linker ($(GGS)_4$) and C-terminal His-tag.

```
MADTRIGVTIYKAAAMVSKGEELFTGVVPILVELDGDVNGHKFSVSGEGEGDATYGKLTLKFICTTGKLPVPWPTLVTT
LSWGVQCFARYPDHMKQHDFFKSAMPEGYVQERTIFFKDDGNYKTRAEVKFEGDTLVNRIELKGIDFKEDGNILGHKLE
YNYFSDNVYITADKQKNGIKANFKIRHNIEDGGVQLADHYQQNTPIGDGPVLLPDNHYLSTQSKLSKDPNEKRDHMVLL
EFVTAAGITLGMDELYGSGGSGGSGGSGGSPGDNFMSVVRKAIEQDAKAAPDVQLLMNDSQNDQSKQNDQIDVLLAKGV
KALAINLVDPAAAGTVIEKARGQNVPVVFFNKEPSRKALDSYDKAYYVGTDSKESGIIQGDLIAKHWAANQGWDLNKDG
QIQFVLLKGEPGHPDAEARTTYVIKELNDKGIKTEQLQLDTAMWDTAQAKDKMDAWLSGPNANKIEVVIANNDAMAMGA
VEALKAHNKSSIPVFGVDALPEALALVKSGALAGTVLNDANNQAKATFDLAKNLADGKGAADGTNWKIDNKVVRVPYVG
VDKDNLAEFSKKEFVDGGMVSKGEELFTGVVPILVELDGDVNGHKFSVSGEGEGDATYGKLTLKLICTTGKLPVPWPTL
VTTLGYGLQCFARYPDHMKQHDFFKSAMPEGYVQERTIFFKDDGNYKTRAEVKFEGDTLVNRIELKGIDFKEDGNILGH
KLEYNYNSHNVYITADKQKNGIKANFKIRHNIEDGGVQLADHYQQNTPIGDGPVLLPDNHYLSYQSALSKDPNEKRDHM
VLLEFVTAAGITLGMDELYKHHHHHH
```

# S2 Simulation of Biosensors with Fluorescent Proteins

We simulated sensor construct no. 2 in the simulation framework of structure based models (SBMs). SBMs are a coarse-grained approach commonly applied in protein folding [2, 3] which provide access to long time scales with modest computational demands. For the simulations, a starting structure of the whole construct in atomic detail was required. The first steps described here are the generation of this sensor structure. In addition, we provide a systematic protocol to obtain structures and parameters for the whole system. Then we give an overview on the simulation protocol and analyze the simulations. Finally, we consider the flexibility of the fluorescent proteins (FPs) in the sensor construct.

## S2.1 Sensor Sequence for Simulation

The sequence of the investigated sensor starts with residues 1 to 11 of MglB and three alanine residues which are inserted as a restriction site to enable proper folding of the proteins. The donor FP is then attached to residue 12 of MglB via a flexible $(GGS)_4$-linker and two restriction sites, each consisting of two amino acids (`GS(GGS)`$_4$`PG`). The acceptor FP is attached to the C-terminus of MglB via a restriction site with sequence `EFVDGG`. In the simulations we omitted parts of the N- and C-termini of the construct where no resolved structures were available. However, N- and C-termini typically express large structurally mobility or “floppyness” and they should not effect the results. The full sensor sequence used in experiments can be found in Sec. S1 (B), a schematic depiction of the sequence can be found in the main text (see Fig. 3a).

## S2.2 Protein Structures

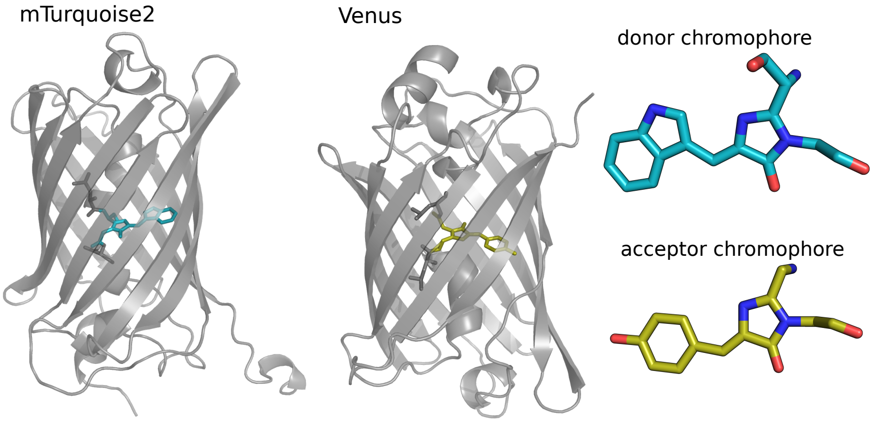


*Figure S2.1: The two FPs mTurquoise2 and Venus along with their respective chromophores of donor FP (cyan) and acceptor FP (yellow).*

For both FPs, PDB structures are available. Fig. S2.1 shows the structures of the donor FP mTurquoise2 (PDB: 3ZTF [4]) and the acceptor FP Venus (PDB: 1MYW [5]) along with the two chromophores.

For MglB two PDB structures are available, i.e. the structure with glucose-bound ($MglB^{+G}$, PDB: 2FVY [6]) and the apo open form without glucose ($MglB^{-G}$, PDB: 2FW0 [6]). In both structures, the first and the last three residues are not resolved. In the simulations, the first residue was omitted and the last three residues were included in the subsequent structure of the

restriction site. According to the sequence used for the glucose sensor, residues 12 and 13 were deleted in the MglB structures.

## S2.3 Protein Parameters

The simulations in SBMs also require topologies, i.e. connectivity and force-field parametrization, of the structures. The topologies for all proteins were generated with `eSBMTools` [7]. For the chromophores in the FPs we generated topologies in the same way as was done for the FRET dyes in [8]. Then, the chromophores were included into the topology generation in `eSBMTools`.

As SBMs do not use physical temperatures, this is another parameter to be determined. In the following, temperatures are given in the `GROMACS` reduced temperature unit which for SBMs does not directly correspond to physical temperatures.

The atom masses for both FPs were set to $m_{\mathrm{FP}} = 0.2$ to accelerate the dynamics in the simulations, as was done for the dyes in [8]. The temperature for the two FPs was determined by comparison of regular MD simulations using the AMBER99 force field [9] with SBM simulations in the same way as described in [10]. The resulting temperatures are $T = 70$ for both, the donor and acceptor FP. For MglB, temperature comparison simulations are not reasonable as the ligand is not straightforward to parametrize and include into regular MD simulations. In this case we used the same temperature as for the FPs, which is a reasonable temperature to simulate the structural fluctuations of folded proteins in SBMs.

## S2.4 Linker Structures

As there is no three-dimensional structures of the linker and little information about its behavior available, the linker structure provides a particular challenge. Also the structures of the restriction sites are not known and the three C-terminal residues in MglB not resolved. To incorporate these regions into the simulations, we generated structures of the sequences shown in Tab. S1 in the following way.

*Table S1: Sequences modeled for the N-terminal linker and the C-terminal restriction site of the sensor. The last three residues of MglB (green) are included in the sequence as they are not resolved in the structure. The flexible (GGS)$_4$-linker and the restriction sites are colored in purple and black, respectively.*

| | Sequence |
|---|---|
| N-terminus | GS(GGS)$_4$PG |
| C-terminus | SKKEFVDCC |

First, we built the respective sequence in `pymol` [11] as extended amino acid chain without any secondary structure. We relaxed each structure by simulating it in an all-atom AMBER99 force field [9] with explicit water for 100 ns at a temperature of 295K.

As we needed to attach the N- and C-termini of the linkers to the protein structures, the respective atoms needed to be accessible. While this was the case for the structure of the C-terminal restriction site, the relaxed structure of the N-terminal linker was too compact to merge with the protein structure. The contained (GGS)$_4$-linker is known to have a random coil like structure and being flexible [12]. To obtain a good starting point for the generation of the merged structures later, we performed AMBER99 simulations with a pulling force. We pulled at N- and C-terminus of the N-terminal linker with a small force to make the termini accessible.

In the merged system the proteins are attached to the termini and apply a force on the linker, so this seems to be a valid procedure. From this simulation we extracted four different structures from different points in the simulations, and used them for the next step.

## S2.5 Linker Parameters

The dynamic linker behavior can be measured experimentally only indirectly but influences the dynamics of the system. Hence, to obtain a reference for the linker flexibility, we performed further simulations, starting from the last structures of the AMBER99 relaxation and the AMBER99 pulling force runs. We simulated the two structures of N-terminal linker and C-terminal restriction site for 500 ns in an AMBER99 force field with explicit water, respectively. Additionally, we simulated the same structures in an SBM with temperatures ranging from $T = 40$ to $T = 150$. An important characteristic of a linker in this system is its end-to-end distance, as it plays a huge role in the distance between the two FPs. So we compared the means and standard deviations of the end-to-end distances from AMBER99 and SBM simulations for all structures.

The C-terminal restriction site should be rather rigid, so we checked if a choice of $T = 70$ as determined for the proteins is valid. There was no big change in the mean or standard deviation of the end-to-end distance as a function of $T$ in the SBM simulations and the values were close to the values from the AMBER99 simulation. Given the limited information available about these structures, assuming a temperature of $T = 70$ seems to be an adequate starting point allowing for reasonable flexibility.

For the N-terminal linker, attaching the completely relaxed structure to the proteins was not possible due to inwardly rotated termini. Simulations of the strongly extended structures showed an immoderately high mean distance over all temperatures. In this case we chose an only slightly elongated structure, where the termini were accessible, and which had the best agreement in terms of end-to-end distance at around $T = 140$. In accordance with the N-terminal linker containing the flexible linker region $(GGS)_4$, we chose a temperature of $T = 140$ for the $(GGS)_4$-linker and the protein temperature of $T = 70$ for the restriction site residues in all further simulations.

## S2.6 Merging of Sensing Protein and Fluorescent Proteins

We assumed that still all residues, including residues 1 to 11, fold into the given MglB structure (for validation see below).

We start with the sensing protein MglB and used the structure of the glucose-bound state, as the transition from the closed to the open conformation with attached FPs was easier than vice versa. After removing residues 12 and 13 as done in experiments we attached the N-terminal linker and C-terminal amino acids corresponding to the genetic restriction site with the algorithm previously described in [8]. The C-terminus of the N-terminal linker was attached to residue 14 and the N-terminus of the C-terminal linker to the last structurally resolved residue (residue 303) of MglB. Different than in [8] here we tested all possible orientations of the linkers in respect to MglB using a set of angles $\alpha, \theta, \phi$ in discrete steps. We checked for steric clashes and saved all sterically possible conformations.

To reduce computational costs later we reduced the number of considered structures by filtering for similar structures. We calculated the mutual RMSD between all constructed structures with both linkers and discarded every structure which differed less than $\text{RMSD}_{\text{cutoff}} = 0.1\,\text{nm}$ from any of the already chosen structures.

With the remaining structures we proceeded by attaching the donor FP and the acceptor FP in the same way. The C-terminus of the donor FP was attached to the N-terminus of the

N-terminal linker and the N-terminus of the acceptor FP to the C-terminus of the C-terminal linker. Again, we filtered the resulting structures and discarded structures which differed less than $\mathrm{RMSD}_{\mathrm{cutoff,\,FP}} = 1.2\,\mathrm{nm}$ from the already chosen structures. We proceeded with the resulting 127 structures. As a next step we attached the three missing alanine residues at residue 11 of MglB using `pymol` [11] and subsequently performed an energy minimization for 5000 steps to get rid of possible clashes caused by this insertion.

The last step consisted of two short SBM simulations to connect the alanine residues with the N-terminus of the donor FP. To enable inclusion of a regular bond potential in the final structure, the target distance for the respective atoms was set to $d_{\mathrm{target}} = 0.14\,\mathrm{nm}$. In these simulations we set the temperature of the three alanine residues and the N-terminal linker to $T = 140$ to allow them to flexibly adjust to the rest of the structure.

We pulled the two atoms together in two consecutive steps to avoid large forces. This was done by introducing a soft contact potential between the N-terminus of the donor FP and the carbon atom of the third alanine with a minimum at the target distance, a width of $\sigma = 2\,\mathrm{nm}$, and a force constant of $K_{\mathrm{c}} = 40\,\epsilon$ and simulation for 1000 ps in the SBM (see also below). The structure of MglB in the construct stayed intact, confirmed by checking the RMSD of MglB. Then the structure with the distance of the respective atoms closest to the target distance was extracted.

We proceeded by introducing a weak bond potential with a minimum at the target distance and a force constant of $K_{\mathrm{b}} = 80\,\epsilon/\mathrm{nm}^2$. We simulated the system for 500 ps in the SBM. For the rare case the distance did not reach $d < 0.3\,\mathrm{nm}$, the desired bond might be sterically inhibited and the structure was discarded. Otherwise, the structure with the distance closest to the target distance was extracted and used for further simulations.

This procedure resulted in a wide ensemble of possible structures for the sensor. To narrow the conformational space for the final simulations, we fitted the resulting structures to experimental SAXS data of the sensor in the glucose-bound state using `CRYSOL` [13]. As starting structures for the simulation we chose the four best fitting structures with the lowest $\chi^2$ value. These structures are denoted with sensor 2-1, sensor 2-2, etc. according to rank in the fitting to the experimental SAXS data (best fitting model: 2-1, second best: 2-2, etc.). The structures are shown in the main text (see Fig. 3c).

## S2.7 Simulation Protocol for Fluorescent Proteins

All simulations of the sensor construct were performed with `GROMACS v4.5.4` [14] with the extension for Gaussian contact potentials [15], Langevin dynamics and the following SBM potential

(see also [15], [8]):

$$
\begin{aligned}
V_{\mathrm{SBM}} \quad &= \sum_{\text{bonds}} K_{\mathrm{b}}(r-r_0)^2 + \sum_{\text{angles}} K_{\mathrm{a}}(\theta-\theta_0)^2 \\
&+ \sum_{\substack{\text{improper}\\ \text{dihedrals}}} K_{\mathrm{i}}(\chi-\chi_0)^2 \\
&+ \sum_{\substack{\text{proper}\\ \text{dihedrals}}} K_{\mathrm{d}} \left[ [1-\cos(\phi-\phi_0)] + \frac{1}{2}\,[1-\cos(3(\phi-\phi_0))] \right] \\
&+ \sum_{\text{contacts}} K_{\mathrm{c}} C_{\mathrm{G}}(r_{ij}, r_0^{ij}) \\
&+ \sum_{\substack{\text{non-native}\\ \text{contacts}}} K_{\mathrm{nc}} \left(\frac{\tilde{\sigma}}{r_{ij}}\right)^{12} . \qquad (1)
\end{aligned}
$$

The SBM potential includes harmonic potentials for bonds, angles, and improper dihedral angles. The native structure is employed as the ground state with native bond lengths $r_0$, native angles $\theta_0$, native improper dihedral angles $\chi_0$, and native proper dihedral angles $\phi_0$. The contact potential $C_{\mathrm{G}}(r_{ij}, r_0^{ij})$ introduces attractive interactions for atom pairs forming contacts in the native state. Additionally, a repulsive term is added for all possible atom pairs to account for the excluded volume. Here, $r_0^{ij}$ and $r_{ij}$ denote the native and the actual distance of the atom pair $(i, j)$. $\tilde{\sigma}$ represents the excluded volume for Pauli repulsion with $\tilde{\sigma} = 0.25\,\mathrm{nm}$. The force constants were set to $K_{\mathrm{b}} = 20000\,\epsilon/\mathrm{nm}^2$, $K_{\mathrm{a}} = 40\,\epsilon/\mathrm{deg}$, $K_{\mathrm{i}} = 40\,\epsilon/\mathrm{deg}$, and $K_{\mathrm{nc}} = 0.01\,\epsilon$, where deg refers to degree and $\epsilon$ is the reduced energy unit used in these types of models [16].

The Gaussian contact potential is given by [17, 15]:

$$
\begin{aligned}
C_{\mathrm{G}}(r_{ij}, r_0^{ij}) \quad &= \left(1 + \left(\frac{\tilde{\sigma}}{r_{ij}}\right)^{12}\right) \\
&\times \left(1 - \exp\left[-\frac{(r_{ij}-r_0^{ij})^2}{2\sigma^2}\right]\right) - 1\,, \qquad (2)
\end{aligned}
$$

with $\sigma^2 = (r_0^{ij})^2/(50 \ln 2)$ for each native contact pair $(i, j)$. Between the different parts of the sensor construct (proteins, linker, restriction site) no contacts were included, except the contacts between the first eleven residues and the rest of MglB.

For the simulation, the time step and the temperature coupling constant were set to $\Delta t_{\mathrm{SBM}} = 0.5\,\mathrm{fs}$ and $\tau_T = 0.1\,\mathrm{ps}$, respectively. For each starting structure, we simulated a total time of $t_{\mathrm{SBM}} = 1000\,\mathrm{ns}$. In the simulation, the system was separated into different temperature groups, where the temperature of the $(\mathrm{GGS})_4$-linker was set to $T = 140$ to provide flexibility, while the rest of the system (proteins and restriction sites) was coupled to a temperature of $T = 70$.

SBMs do not have an inherent time scale, so we adjusted the time scale by comparison of rotational correlation times between SBM simulations and experimental values. The rotational correlation time of the free green fluorescent protein is often estimated with $\tau_{\mathrm{rot}} \sim 20\,\mathrm{ns}$. For the free donor FP we measured $\tau_{\mathrm{rot}} = 20.85\,\mathrm{ns}$. As the rotational correlation time in free solution is mainly dependent on the friction with the solvent, a comparison of SBM simulations of a freely diffusing FP with the experimentally measured values should yield a friction which mimics the physical behavior. We performed simulations for $t_{\mathrm{SBM}} = 50\,\mathrm{ns}$ with free donor FP and acceptor FP including the chromophores. We calculated the free rotational correlation times using the

time dependent fluorescence anisotropy [18]

$$r(t) = r_0 \left\langle P_2 \left[ \widehat{\boldsymbol{\mu}}(s) \cdot \widehat{\boldsymbol{\mu}}(s+t) \right] \right\rangle_s \,, \tag{3}$$

where $r_0$ is the fundamental anisotropy and $P_2$ the second-order Legendre polynomial given by $P_2(x) = \frac{1}{2}(3x^2 - 1)$. With the assumption of collinear transition dipole moments, the fundamental anisotropy is given by $r_0 = 0.4$. We fitted this with one exponential function with the relation [19]

$$r(t) = r_0 \exp\left[ -\frac{t}{\tau_{\mathrm{rot}}} \right] . \tag{4}$$

Then we used the ratio between experimental and simulated values as conversion factor to adjust the simulation time scale. This allows us to give estimates for the rotational correlation times of the FPs attached to MglB.

For simulation of MglB$^{\text{-G}}$ with the same configuration of FPs, we used the same starting structure, but replaced the parts of the force field (bond, angle, dihedral angle, and contact parameters) belonging to MglB$^{\text{+G}}$ with the respective values of MglB$^{\text{-G}}$. Figs. S2.2 and S2.3 show that the conformation changed accordingly and both conformations (MglB$^{\text{+G}}$ and MglB$^{\text{-G}}$) stayed stable during the simulations.

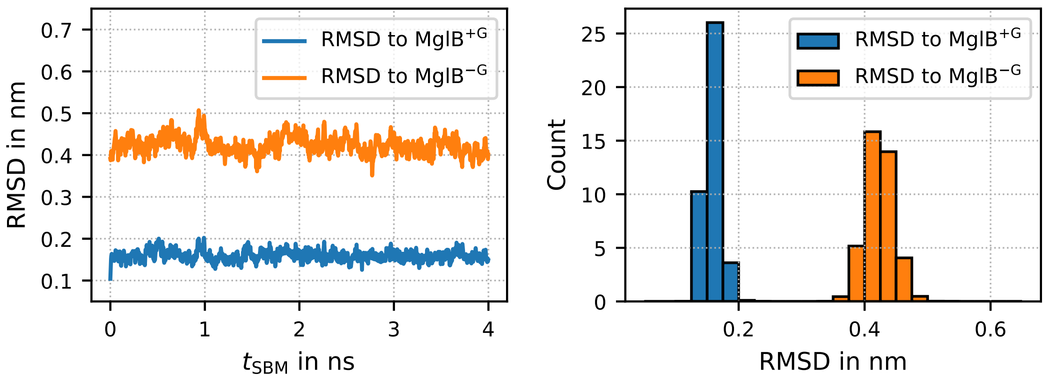


*Figure S2.2: Exemplary RMSD values from simulation of the sensor with MglB in its glucose-bound state. The RMSD values of MglB with reference to MglB$^{+G}$ (blue) and MglB$^{-G}$ (orange) are shown for the start of the simulation (left) and as distributions for the entire simulation (right). The conformation is stable over the entire simulation.*

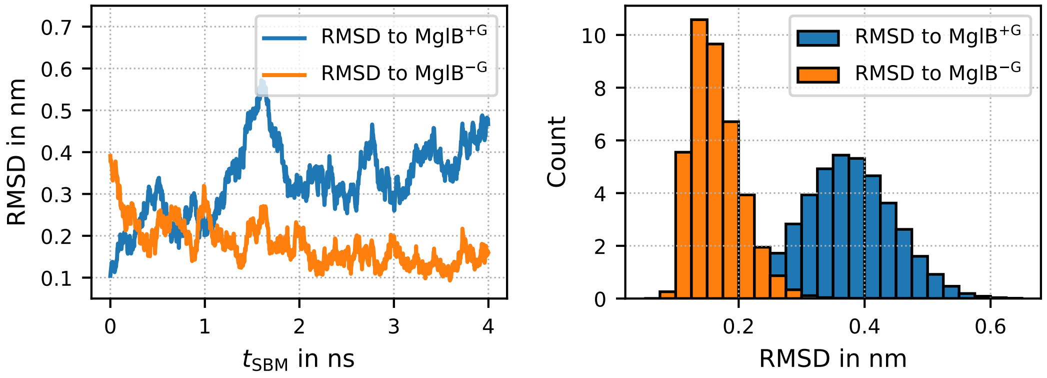


*Figure S2.3: Exemplary RMSD values from simulation of the sensor with MglB in its glucose-free state. The RMSD values of MglB with reference to MglB$^{+G}$ (blue) and MglB$^{-G}$ (orange) are shown for the start of the simulation (left) and as distributions for the entire simulation (right). A transition from the starting structure (MglB$^{+G}$) to the target structure (MglB$^{-G}$) can be seen directly at the beginning of the simulation.*

## S2.8 Analysis of Simulations

To obtain a larger structural ensemble and investigate the dynamics of the sensor, we performed SBM simulations of the sensor construct with the four selected starting structures. We performed simulations with MglB in the glucose-bound state and in the glucose-free state, respectively. The simulations cover $t_{\mathrm{SBM}} = 1000\,\mathrm{ns}$, which represents approximately $100\,\mu\mathrm{s}$ on the physical time scale.

Using `CRYSOL` [13], we generated SAXS intensity profiles of structures distributed evenly over the entire simulation and averaged the intensities to obtain mean intensity curves for each simulation.

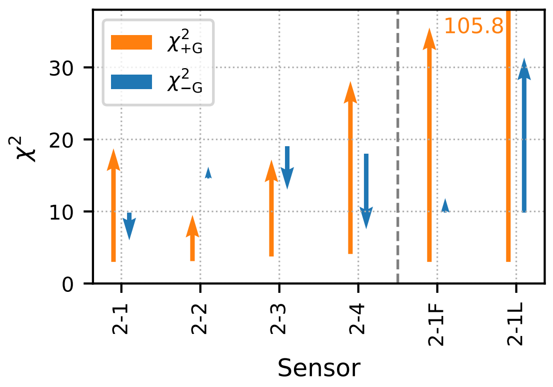


*Figure S2.4: Change in $\chi^2$ from starting structure to mean intensity of the simulation with respect to the experimental SAXS data, respectively. The arrows start at the $\chi^2$ value of the starting structure and end at the $\chi^2$ value of the mean intensity. For the different starting structures, the respective values are depicted for MglB$^{+G}$ (orange) and MglB$^{-G}$ (blue) states. The endpoint of the arrow exceeding the figure is denoted alongside. In addition to the four mentioned starting structures (2-1, 2-2, 2-3, 2-4), also two variants are depicted (2-1F, 2-1L, see text).*

Fig. S2.4 depicts the change in $\chi^2$ calculated from the fit to the experimental SAXS data from starting structure to mean intensity curve of the simulation for all sensor simulations. The simulations of the glucose-free state are in good overall agreement with the experimental data. However, for almost all starting structures the simulations of the glucose-bound state diverge from the experimental data.

To test different hypotheses, we simulated two variants of sensor construct no. 2. In Fig. S2.4 they are denoted as sensor 2-1F and sensor 2-1L. Both simulations used the same starting structure as sensor 2-1, but differed in contacts and flexibility. In sensor 2-1F, in addition to the flexible $(GGS)_4$-linker, all restriction sites were kept flexible. As the restriction sites were added artificially to the structure and their structures were not known, this could be a valid model. However, the high $\chi^2$ values in the simulation of sensor 2-1F indicate that its agreement with experimental data is worse than for the other simulations of sensor construct no. 2. The original parametrization chosen for the restriction sites seems to be preferable.

To test if MglB refolds completely including residues 1 to 11 with the donor FP inserted, we simulated sensor 2-1L with no contacts between residues 1 to 11 and the remaining part of MglB. In the simulation, the N-terminal residues could loosen themselves from the rest of the structure accordingly. Sensor 2-1L clearly shows larger divergence from the SAXS data than sensor 2-1, making this scenario unlikely.

The simulations provide direct access to the inter-FP distances $R_{\mathrm{DA}}$ and orientation factors $\kappa^2$ of the system. We extracted $R_{\mathrm{DA}}$ as the distance of the centers of mass of the two chromophores of donor and acceptor.

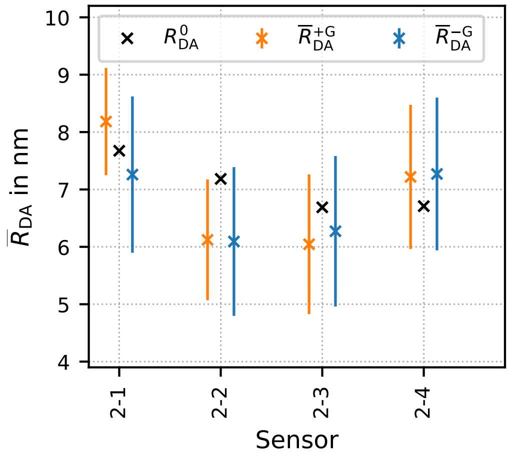


*Figure S2.5: Distance distributions for the different simulations. The mean distances $\overline{R_{DA}}$ (crosses) and the standard deviations $\sigma_{DA}$ of the distribution (error bars) are depicted for simulations with glucose (+G, orange) and without glucose (-G, blue). The inter-FP distances $R^0_{DA}$ of the respective starting structures are shown as black crosses.*

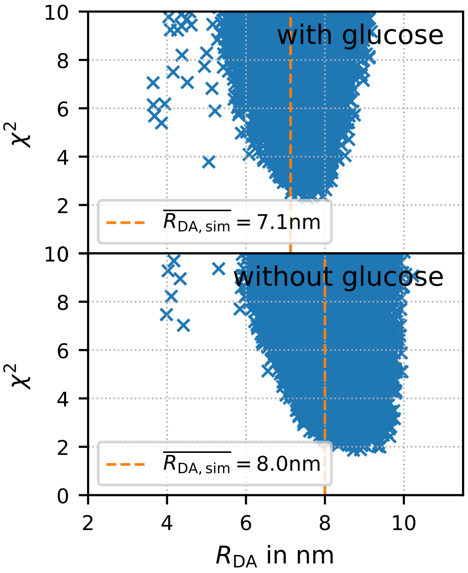


*Figure S2.6: 2D-plots of simulation results. In these graphs each simulated structure is represented by a blue cross characterizing the corresponding structure by its $R_{DA}$ values and the deviation from the corresponding SAXS data in terms of $\chi^2$ values. Feasibe agreement of the simulation results with experimental SAXS data is achieved for $\chi^2 < 10$. For these structures a mean $R_{DA,\ sim}$ value was calculated which is represented by the orange broken line.*

As evident from Figs. S2.5 and S2.6, the distance distributions of the simulations without glucose are always broader than that of the simulations with glucose. The FPs are more flexible in this glucose-free state. However, the distributions of the different simulations vary noticeably.

## S2.9 Flexibility of Fluorescent Proteins

To compare the flexibility of both FPs in the sensor, we calculated the fluorescence anisotropy and the rotational correlation times $\tau_{\text{rot}}$ of the FPs bound to MglB. By fitting the simulated trajectory to MglB, we could observe the FP motion independently of the overall rotation of the system. Two exemplary fits are shown in the main text (see Fig. 4a). Due to the restriction of the motions, we used the "wobbling-in-a-cone" model [20]. This model assumes that the transition dipole moments of the chromophores move freely in a cone. Then the anisotropy decay can be described with

$$r(t) = r_0 \left[ (1 - A) \exp\left[ -\frac{t}{\tau_{\text{rot}}} \right] + A \right], \quad (5)$$

where $r_0$ denotes the fundamental anisotropy and $A$ defines a measure of the rotational restriction and is related to the angle of the cone.

Converting the resulting values for rotational correlation times from the different simulations with the factor determined to adjust the time scale before, results in a time regime of $\tau_{\text{rot}} = 190 - 290\,\text{ns}$ on the physical time scale.

Considering the parameter $A$ representing the spatial restriction, it turns out that the acceptor chromophore is rather free with values of $A = 0.01 - 0.05$. In contrast, the donor chromophore is hindered with values in the range of $A = 0.07 - 0.17$. Furthermore, the donor chromophore always gains flexibility, i.e. $A$ decreases, in the structures without glucose, whereas the change for the acceptor chromophore is negligible. It should be noted that the donor FP is mainly hindered in its rotation about the cylinder axis of the $\beta$-barrel, resulting from its two attachment points to MglB.

## S3 SAXS results

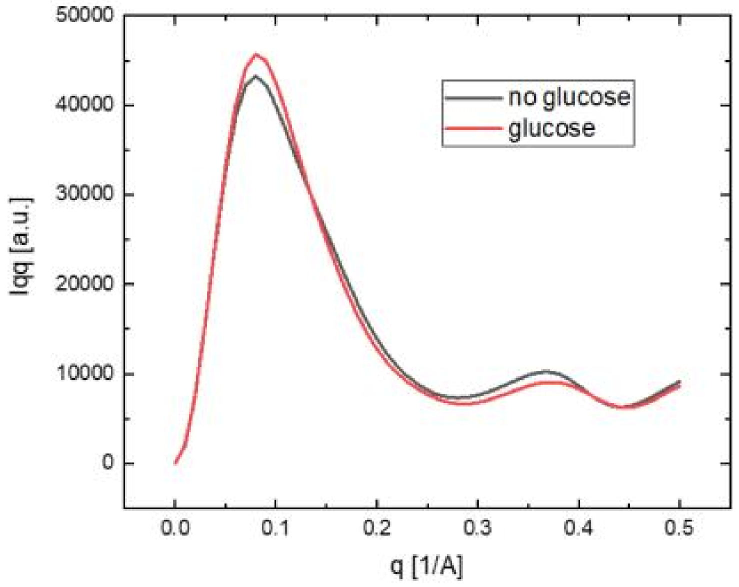


*Figure S3.1: Calculated Kratky plots based on the crystal structure of the glucose-free state (2FW0) and the glucose-bound state (2FWY) [6] of MglB. The resulting $R_G$-values are 22.84 Å and 21.73 Å for the glucose-free and glucose-bound structures, respectively, demonstrating the compaction of MglB upon glucose binding.*

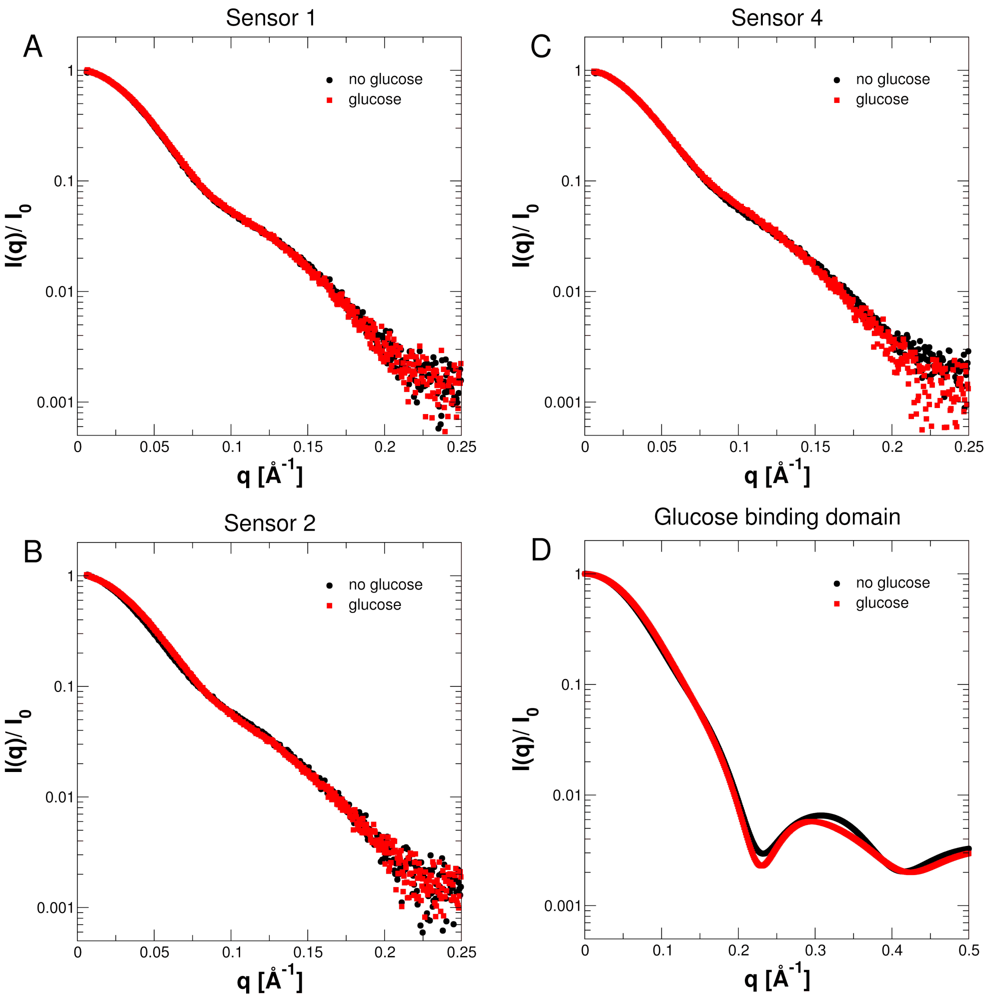


*Figure S3.2: Experimental SAXS data of (A, B, C) the sensor constructs in absence and presence of glucose and (D) calculated SAXS curves based on the crystal structure of the glucose-free state (2FW0) and the glucose-bound (2FWY) of MglB.*

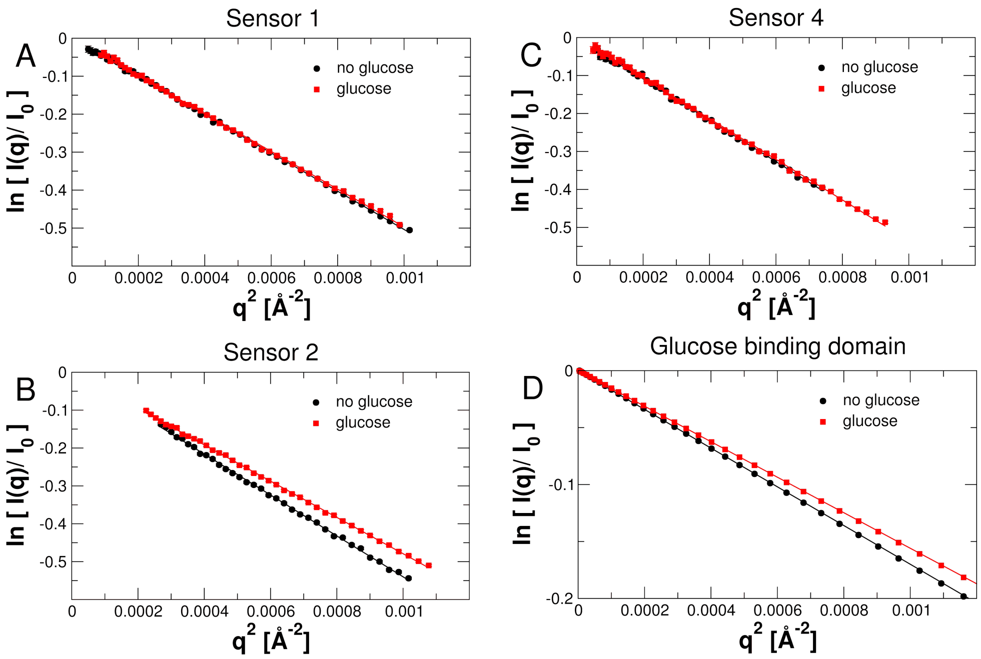


*Figure S3.3: Guinier plots of the experimental SAXS data of (A, B, C) the sensor constructs in absence and presence of glucose and (D) calculated SAXS curves based on the crystal structure of the glucose-free state (2FW0) and the glucose-bound (2FWY) of MglB.*

*Table S2: Parameters determined from experimental SAXS data. *Theoretical values based on PDB IDs (MglB: 2FW0; MglB + Glucose: 2FVY), $^{+}D_{max}$ = maximal distance of the $P(r)$ function = maximal dimension of particle*

| | Sensor 1 | Sensor 1 +Glucose | Sensor 2 | Sensor 2 +Glucose | Sensor 4 | Sensor 4 +Glucose | MglB* | MglB +Glucose* |
|---|---|---|---|---|---|---|---|---|
| $R_G$ (Å) Guinier | 38.87 ± 0.06 | 38.60 ± 0.08 | 40.25 ± 0.11 | 37.91 ± 0.07 | 40.42 ± 0.10 | 40.05 ± 0.08 | 22.77 | 21.76 |
| $R_{G,\text{ rec}}$ (Å) $P(r)$ rec. space | 39.11 | 38.11 | 41.08 | 38.04 | 40.92 | 40.79 | 22.74 | 21.74 |
| $R_{G,\text{ real}}$ (Å) $P(r)$ real space | 39.21 | 38.11 | 41.25 | 38.16 | 40.97 | 40.83 | 23.27 | 21.73 |
| $D_{\max}$ (Å)$^{+}$ | 133.3 | 142.7 | 139.7 | 140.1 | 139.3 | 139.8 | 72.62 | 72.30 |
| $M_m$ (kDa) Porod | 89.6 | 85.7 | 83.9 | 78.7 | 85.7 | 79.2 | 33.3 | 33.3 |
| $M_m$ (kDa) Bay. Interf. | 94.2 ± 5.0 | 94.2 ± 7.3 | 94.2 ± 5.0 | 91.2 ± 6.8 | 94.2 ± 9.9 | 94.2 ± 4.5 | 31.7 ± 2.4 | 31.7 ± 3.1 |
| $M_m$ (kDa) Sequence | 86.5 | 86.5 | 89.5 | 89.5 | 83.8 | 83.8 | 31.9 | 31.9 |

## References


[1] H. Höfig, J. Otten, V. Steffen, M. Pohl, A. J. Boersma, and J. Fitter, "Genetically Encoded Förster Resonance Energy Transfer-Based Biosensors Studied on the Single-Molecule Level," *ACS Sensors*, vol. 3, pp. 1462–1470, aug 2018.

[2] L. L. Chavez, J. N. Onuchic, and C. Clementi, "Quantifying the roughness on the free energy landscape: entropic bottlenecks and protein folding rates.," *Journal of the American Chemical Society*, vol. 126, pp. 8426–32, jul 2004.

[3] C. Sinner, B. Lutz, A. Verma, and A. Schug, "Revealing the global map of protein folding space by large-scale simulations," *The Journal of Chemical Physics*, vol. 143, p. 243154, dec 2015.

[4] A. Rekas, J.-R. Alattia, T. Nagai, A. Miyawaki, and M. Ikura, "Crystal Structure of Venus, a Yellow Fluorescent Protein with Improved Maturation and Reduced Environmental Sensitivity," *Journal of Biological Chemistry*, vol. 277, pp. 50573–50578, dec 2002.

[5] J. Goedhart, D. von Stetten, M. Noirclerc-Savoye, M. Lelimousin, L. Joosen, M. A. Hink, L. van Weeren, T. W. Gadella, and A. Royant, "Structure-guided evolution of cyan fluorescent proteins towards a quantum yield of 93%," *Nature Communications*, vol. 3, p. 751, jan 2012.

[6] M. J. Borrok, L. L. Kiessling, and K. T. Forest, "Conformational changes of glucose/galactose-binding protein illuminated by open, unliganded, and ultra-high-resolution ligand-bound structures," *Protein Science*, vol. 16, pp. 1032–1041, jun 2007.

[7] B. Lutz, C. Sinner, G. Heuermann, A. Verma, and A. Schug, "eSBMTools 1.0: enhanced native structure-based modeling tools," *Bioinformatics*, vol. 29, pp. 2795–2796, nov 2013.

[8] I. Reinartz, C. Sinner, D. Nettels, B. Stucki-Buchli, F. Stockmar, P. T. Panek, C. R. Jacob, G. U. Nienhaus, B. Schuler, and A. Schug, "Simulation of FRET dyes allows quantitative comparison against experimental data," *The Journal of Chemical Physics*, vol. 148, p. 123321, mar 2018.

[9] J. Wang, P. Cieplak, and P. A. Kollman, "How well does a restrained electrostatic potential (RESP) model perform in calculating conformational energies of organic and biological molecules?," *Journal of Computational Chemistry*, vol. 21, pp. 1049–1074, sep 2000.

[10] B. Lutz, M. Faber, A. Verma, S. Klumpp, and A. Schug, "Computational Analysis of Co-Transcriptional Riboswitch Folding," *Biophysical Journal*, vol. 106, p. 284a, jan 2014.

[11] Schrödinger, LLC, "The PyMOL molecular graphics system, version 1.8." November 2015.

[12] E. M. W. M. van Dongen, T. H. Evers, L. M. Dekkers, E. W. Meijer, L. W. J. Klomp, and M. Merkx, "Variation of Linker Length in Ratiometric Fluorescent Sensor Proteins Allows Rational Tuning of Zn(II) Affinity in the Picomolar to Femtomolar Range," *Journal of the American Chemical Society*, vol. 129, pp. 3494–3495, mar 2007.

[13] D. Svergun, C. Barberato, and M. H. Koch, "CRYSOL - A program to evaluate X-ray solution scattering of biological macromolecules from atomic coordinates," *Journal of Applied Crystallography*, vol. 28, no. 6, pp. 768–773, 1995.

[14] D. van der Spoel, E. Lindahl, B. Hess, A. R. van Buuren, E. Apol, P. J. Meulenhoff, D. P. Tieleman, A. L. T. M. Sijbers, K. A. Feenstra, R. van Drunen, and H. J. C. Berendsen, *Gromacs User Manual version 4.5.6.* URL: http://www.gromacs.org, 2010.

[15] J. K. Noel, A. Schug, A. Verma, W. Wenzel, A. E. Garcia, and J. N. Onuchic, "Mirror Images as Naturally Competing Conformations in Protein Folding," *The Journal of Physical Chemistry B*, vol. 116, pp. 6880–6888, jun 2012.

[16] J. K. Noel, P. C. Whitford, K. Y. Sanbonmatsu, and J. N. Onuchic, "SMOG@ctbp: simplified deployment of structure-based models in GROMACS," *Nucleic Acids Research*, vol. 38, pp. W657–W661, jul 2010.

[17] H. Lammert, A. Schug, and J. N. Onuchic, "Robustness and generalization of structure-based models for protein folding and function," *Proteins: Structure, Function, and Bioinformatics*, vol. 77, pp. 881–891, dec 2009.

[18] G. Lipari and A. Szabo, "Effect of librational motion on fluorescence depolarization and nuclear magnetic resonance relaxation in macromolecules and membranes," *Biophysical Journal*, vol. 30, pp. 489–506, jun 1980.

[19] J. R. Lakowicz, *Principles of Fluorescence Spectroscopy.* Springer US, 2006.

[20] K. Kinosita, S. Kawato, and A. Ikegami, "A theory of fluorescence polarization decay in membranes," *Biophysical Journal*, vol. 20, pp. 289–305, dec 1977.
